# Optimization of Y and T-shaped microchannels for liquid–liquid extraction

**DOI:** 10.1038/s41598-023-46333-3

**Published:** 2023-11-12

**Authors:** Negah Morshedaski, Farshad Raji, Ahmad Rahbar-Kelishami

**Affiliations:** 1 Research Lab for Advanced Separation Processes, Faculty of Chemical, Petroleum and Gas Engineering, Iran University of Science and Technology (IUST), Narmak, Tehran, Iran; 2 Department of Chemical and Petroleum Engineering, Sharif University of Technology, Tehran, Iran

**Keywords:** Environmental sciences, Chemistry

## Abstract

Solvent extraction on a micro-scale has received much attention due to its advantages in recent years. The purpose of this research is to compare the inlet geometry of T and Y-shaped microchannels. In this research, solvent extraction of Crystal Violet (CV) was investigated using Di-(2-ethylhexyl) phosphoric acid (D2EHPA) extractor and hexane solvent in Y and T-shaped microchannels with lengths of 4, 6, and 8 cm. The effect of parameters such as inlet geometry, length of microchannels (4–8 cm), dye solution pH (3–11), flow rate (1–1.5 mL/h) and the concentration of CV (25–75 ppm) was investigated. The Results showed that under the same conditions, Y-shaped microchannel performance is better than T-shaped microchannel. pH of dye solution phase, flow rate, inlet CV concentration, and microchannel length were obtained as optimal conditions for extraction, 10.9, 1.1 mL/h, 46.4 ppm, and 7.6 cm, respectively, and the amount of extraction, in this case, was % 97/96 was obtained.

## Introduction

The extraction efficiency can be significantly impacted by the microchannels shape. The microchannel inlet geometry including Y, T, and ψ was further investigated. Today, Dyes are used in a variety of sectors including textile, plastic, leather, paper, cosmetics, and food^1^. The complex aromatic molecular structures that synthetic dyes often have help to increase their stability. Their release into the environment will have harmful effects on the environment and the health of living organisms^2–6^.

Wastewaters from textile and dyeing industries are one of the most problematic wastewaters due to the fact that they contain chemicals, suspended substances, toxic compounds, and dyes (the first pollutant detectable by the human eye). Dyes may significantly affect the light activity of aquatic life by reducing the penetration of light (impairment in the photosynthesis of aquatic plants), the appearance of eutrophication, the increase of suspended matter and the turbidity of waters, and it may also be due to the presence of Aromatics, salts, chlorides, etc. are toxic to aquatic life and cause their death. For this reason, it is necessary and inevitable to remove the color from dye effluents and wastewater^7–9^.

During the last three decades, several physical, chemical, and biological decolorization methods have been reported. Few have been accepted by the textile and paper sectors, nevertheless^10,11^. Aqueous solutions that include dye have been removed using a variety of techniques. For the removal of dye, a variety of commonly used techniques can be used, such as micellar-enhanced ultrafiltration, several oxidation processes, electrochemical degradation, ozone-based processes, photocatalytic degradation, electrocoagulation, nanofiltration (NF), ion exchange membranes, adsorption on activated carbon, and electrocoagulation^1,12–23^.

Solvent extraction or liquid–liquid extraction (LLE) has been one of the most common methods for removing pollutants^24^. Excellent throughput, simplicity of automated operation and scaling up, and high purification are some of the benefits of LLE. However, adopting this approach has run into issues due to the lengthy process and significant sample and solvent consumption. Liquid–liquid extraction in microfluidic systems is a new method to overcome the limitations of the traditional liquid–liquid extraction method due to the low sample and solvent consumption and short penetration time^24^.

Kashid et al. compared the geometry of Y and T and found that the geometry of the microchannel affects the boundaries of the flow regime. When compared to a T-shaped square microchannel, the transition from slug flow to slug-droplet flow occurs at a considerably lower flow rate for both phases in a Y-shaped rectangular microchannel^25^. A similar pattern of activity was seen when slug flow changed to deformed interface flow. It is challenging to identify whether element—inlet connection or hydraulic diameter—is the primary cause of the observed variation, though, because the configurations of the two channels feature various inlet connections (T and Y-shaped) as well as varied hydraulic sizes^26^. Kashid et al. separately investigated the change of flow regime in T-shaped square microchannel and T-shaped trapezoidal microchannel and concluded that the cross-sectional area of the microchannel does not affect the flow regime^25^. Raji et al. also investigated the effect of microchannel entrance angle. They concluded that a 60° angle is better than a 30° and 90° angle. Because at this angle, there is better contact between the aqueous and organic phases at the entrance of the channel. As a result, the extraction efficiency increases^27^.

In this research, the influence of the microchannel inlet geometry and the comparison of Y and T-shaped geometry for the selection of the inlet geometry were investigated. CV as a cationic dye with a molecular formula ${(\mathrm{C}}_{25}{\mathrm{N}}_{3}{\mathrm{H}}_{30}\mathrm{Cl})$ and molecular mass of 407.98 g/mol is widely used in the textile industry. This substance is toxic and carcinogenic and causes discomfort if inhaled. Breathing problems, headache, dizziness, and prolonged exposure to it cause damage to the digestive system. The presence of CV in aquatic systems has a direct impact on human life and aquatic ecology. Infiltration of low light leads to damage to photosynthetic processes and in extreme conditions can lead to irreversible blindness as well as kidney and respiratory failure in people, which makes its removal from water sources necessary. The extraction rate was checked in order to optimize this process. Y-shaped geometry and T-shaped geometry are two consecutive geometries in microchannels, and in this research, the effect of each of these two on the extraction process was investigated.

## Materials and methods

### Materials

The materials used in this experiment include crystal violet as the studied pollutant, Hexane as a solvent, and Di-(2-Ethylhexyl) phosphoric acid (D2EHPA) as an extractant. The details of the properties of crystal violet, D2EHPA, and hexane are given in Table 1. Deionized water was used in all experiments.

**Table 1:** Chemical compounds used in the experiment.

Name	Chemical structure	Formula	Density (g/cm^3^)	Purchased from
Crystal violet		${\mathrm{C}}_{25}{\mathrm{N}}_{3}{\mathrm{H}}_{30}\mathrm{Cl}$	1.19	(Merck Co. Germany)
Hexane		${\mathrm{C}}_{6}{\mathrm{H}}_{14}$	0.66	(Merck Co. Germany)
D2EHPA		${\mathrm{C}}_{16}{\mathrm{H}}_{35}{\mathrm{O}}_{4}\mathrm{P}$	0.97	(Merck Co. Germany)

### Fabrication of microreactor

The microchannels were made of Pyrex glass with a thickness of 4 mm and used the CO_2_-laser engraving method. The width and depth of the microchannels are 400 and 300 microns, respectively. Y-shaped microchannels are made in three sizes of 4, 6, and 8 cm, and T-shaped microchannels are made in 8 cm. In order to thoroughly seal the glasses, the channels were employed by applying the technique of direct connection of the bottom and top glass within the furnace. The depth and width of the channels are determined by the laser energy and by the pulse repetition frequency applied along the channel. Laser cutting can be used for almost any material. Laser engraving makes it possible to produce very clean and creative patterns in a short time, even if they are sometimes difficult to reproduce. However, residual particles can be difficult to remove, which causes contamination and uncertainty in the final device. The main drawback of laser engraving and cutting is that it produces relatively large channels with low precision^21,28^.

### Method of experiments

Microchannels with Y and T-shaped inlet geometries were employed in this study. Using D2EHPA extractant and hexane solvent, the CV extraction process was carried out. CV solution and solvent were injected inside the microchannel using a syringe by a 4SP94-1 syringe pump manufactured by Zist-Rad Company of Iran under different flow rates. Hexane solvent was used as a solvent because it has a lower viscosity than other solvents. Because the high viscosity causes the fluid to not move well in the microchannel and higher pumping is required. The extractant used in this research is D2EHPA, which was used at 10 v/v% with hexane as the organic phase. A microscope model HR3-TRF-P from HUVITZ with a 60× magnification and a camera (Canon EOS-700D) to capture images of the currents in the main channel were used to ensure the stability of the parallel currents in the input, output, and also the interface in the main channel. The extraction efficiency (E %) of CV was calculated using Eq. (1):

1 $$E=\frac{{C}_{in}-{C}_{out}}{{C}_{in}}$$

**Figure 1:**
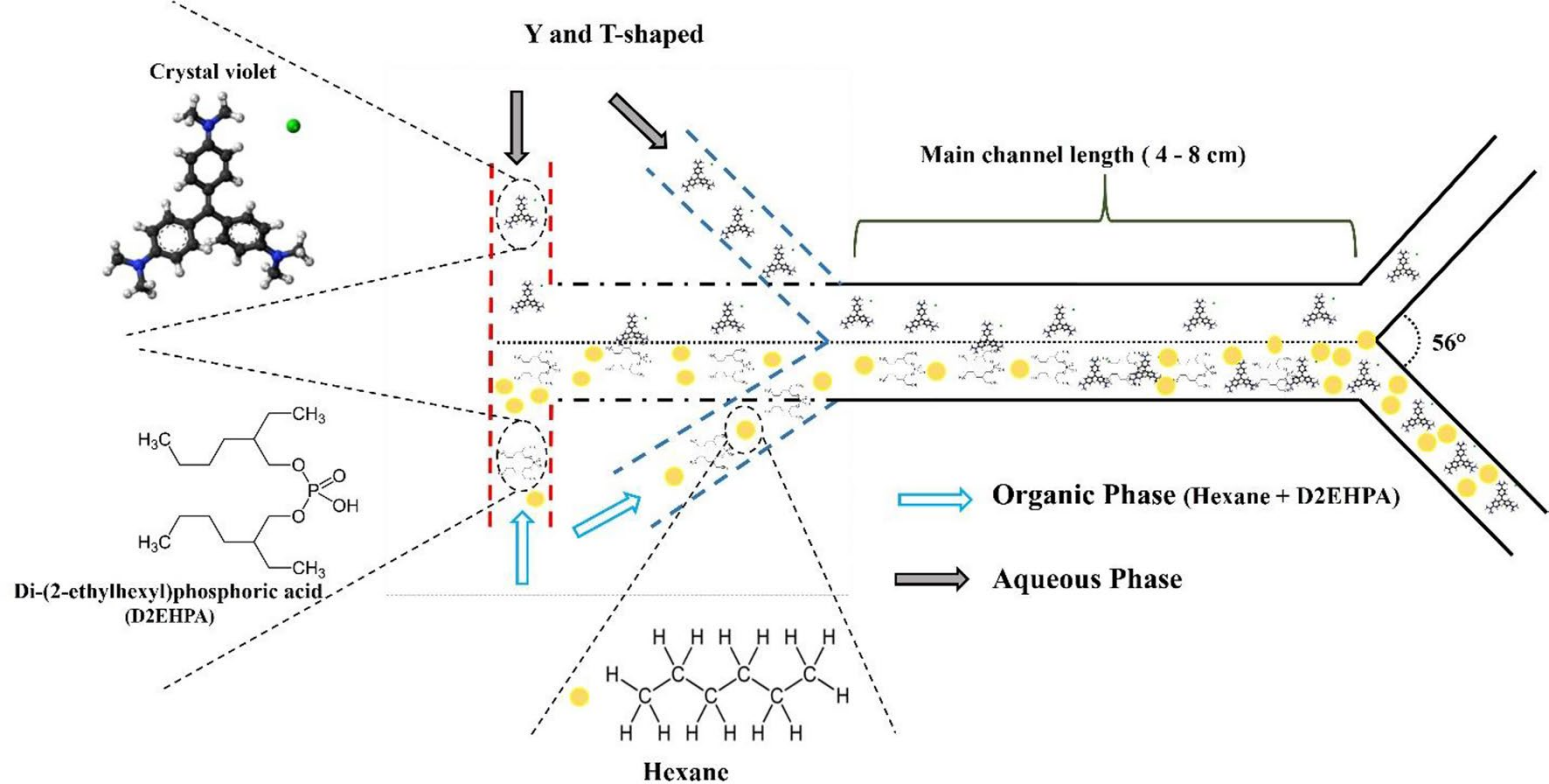
A conceptual schematic of dye extraction in Y and T-shaped microchannel.

C_in_ and C_out_ are the input concentration, and output concentration and E are the percentage of CV extraction, respectively. A diagram of this process is shown in Fig. 1.

### Characterization

A UV–Visible spectrophotometer (Shimadzu UV-1800, Japan) was used to measure the concentration of CV at a maximum wavelength of 581 nm. For the calibration curve, five standard solutions of various concentrations were utilized.

### Design of experiments

Response surface method (RMS) design is used for things like finding the optimal point, troubleshooting process problems, and weak points, and creating a stronger and more resistant process (Robust) against non-controlling factors. Due to the fewer tests required in the design of up to 4 components, Box-Behnken design, or BBD, is one of the response surface design techniques that are of interest. This technique is an incomplete three-level factorial design. When using a BBD design, the number of tests (N) is computed using Eq. (2), where k is the number of components and C_0_ is the typical value of three for the number of central points^29,30^.

2 $$ N = 2k\left( {k - 1} \right) + C_{0} $$

In the present research, 4 factors of dye concentration, pH, flow rate, and microchannel length have been studied. The simultaneous impact of operational factors on several levels was investigated using Design Expert software (version 11.0). The intended response is stated in terms of the effectiveness of CV extraction using the solvent hexane and the extractant D2EHPA. Based on Eq. (3), the quantity of extraction efficiency was estimated. The polynomial equation for CV extraction was determined using Eq. (1), where Y is the predicted response (extraction percentage), α_0_ is a constant coefficient, X_i_ and X_j_ are the values of independent variables, α_i_, α_ii_, and α_ij_ are linear coefficients of the second order and the interaction of two variables^31^.

3 $$Y={\alpha }_{0}+\sum \limits_{i=1}^{3}{\alpha }_{i}{X}_{i}+\sum \limits_{i=1}^{3}{\alpha }_{ii}{X}_{ii}^{2}+\sum \limits_{i=1}^{2}\sum \limits_{i=1}^{3}{\alpha }_{ij}{X}_{i}{X}_{j}$$

The quadratic regression model, which is the relationship between the extraction percentage of CV and operational and geometric parameters, with coded units using the RSM method, is obtained in the form of Eq. (4):

4 $$\begin{aligned} \mathrm{Extraction} & =+84.27 +6.46\mathrm{ A }+4.49\mathrm{ B }+11.38\mathrm{ C }+0.0431\mathrm{ D } \\ & \quad +1.16\mathrm{ AB }-3.89\mathrm{ AC }-0.7575\mathrm{ AD}- 0.9976\mathrm{ BC} \\ & \quad  -1.28\mathrm{ BD }+5.89\mathrm{ CD}-2.71 {\mathrm{A}}^{2}+5.44 {\mathrm{B}}^{2 } \\ & \quad -11.12 {\mathrm{C}}^{2}-4.34 {\mathrm{D}}^{2} \end{aligned}$$

Table 2 displays the experimental outcome and expected value. The model was validated using Analysis of Variance (ANOVA), which was also used to examine the significance of process factors and their impact on the effectiveness of CV extraction. The findings are shown in Table 3. According to Table 3, analysis of variance P-Value less than 0.05 indicates the high accuracy of the presented model. The P parameter indicates the high or low importance of each factor. The predicted value of R^2^ indicates the power of predicting the value by the model. For the adequacy of the model, the difference between the predicted R^2^ and the adjusted R^2^ should be between 0 and 0.2%. In this case, the predicted R^2^ value of 0.9473 is in good agreement with the adjusted R^2^ which is equal to 0.9809.

**Table 2:** Calculated and predicted results for CV extraction.

Run	L (cm)	pH	C0 (ppm)	Flow rate (mL/h)	Experimental	Actual
1	6	3	50	1.25	83.80	85.22
2	4	3	50	1.25	78.01	77.21
3	6	11	50	1.25	93.18	94.19
4	4	11	50	1.25	84.47	83.87
5	8	7	25	1	72.88	71.67
6	8	7	75	1	74.99	74.88
7	8	7	25	1.5	57.84	58.46
8	8	7	75	1.5	83.50	85.23
9	6	7	50	1	81.28	79.89
10	4	7	50	1	70.39	69.96
11	6	7	50	1.5	82.04	79.97
12	4	7	50	1.5	71.66	71.56
13	8	3	25	1.25	68.83	68.20
14	8	11	25	1.25	82.21	81.48
15	8	3	75	1.25	86.46	85.19
16	8	11	75	1.25	95.85	94.48
17	6	7	25	1.25	60.32	61.76
18	4	7	25	1.25	48.21	48.71
19	6	7	75	1.25	84.94	84.53
20	4	7	75	1.25	77.83	79.25
21	8	3	50	1	81.44	82.91
22	8	11	50	1	95.08	96.76
23	8	3	50	1.5	84.24	84.05
24	8	11	50	1.5	92.75	92.76
25	8	7	50	1.25	87.53	88.02
26	8	7	50	1.25	87.78	88.02
27	8	7	50	1.25	88.74	88.02

**Table 3:** ANOVA for the quadratic model for the CV extraction.

Source	Sum of squares	df	Mean of squares	F-value	P-value	
Model	3381.65	14	241.55	96.24	< 0.0001	Significant
A-Length	615.90	1	615.90	245.40	< 0.0001	
B-pH	169.03	1	169.03	67.35	< 0.0001	
C-C0	1088.59	1	1088.59	433.73	< 0.0001	
D-Flow rate	0.0156	1	0.0156	0.0062	0.9348	
AB	9.34	1	9.34	3.72	0.0776	
AC	105.76	1	105.76	42.14	< 0.0001	
AD	4.02	1	4.02	1.60	0.2298	
BC	3.98	1	3.98	1.59	0.2318	
BD	6.58	1	6.58	2.62	0.1315	
CD	138.77	1	138.77	55.29	< 0.0001	
$$ {A}^{2}$$	31.28	1	31.28	12.46	0.0041	
$$ {B}^{2}$$	157.79	1	157.79	62.87	< 0.0001	
$$ {C}^{2}$$	659.30	1	659.30	262.69	< 0.0001	
$$ {D}^{2}$$	100.36	1	100.36	39.99	< 0.0001	
Residual	30.12	12	2.51			
Lack of fit	29.30	10	2.93	7.14	0.1291	Not significant
Pure error	0.8212	2	0.4106			
Core total	3411.77	26				

**Figure 2:**
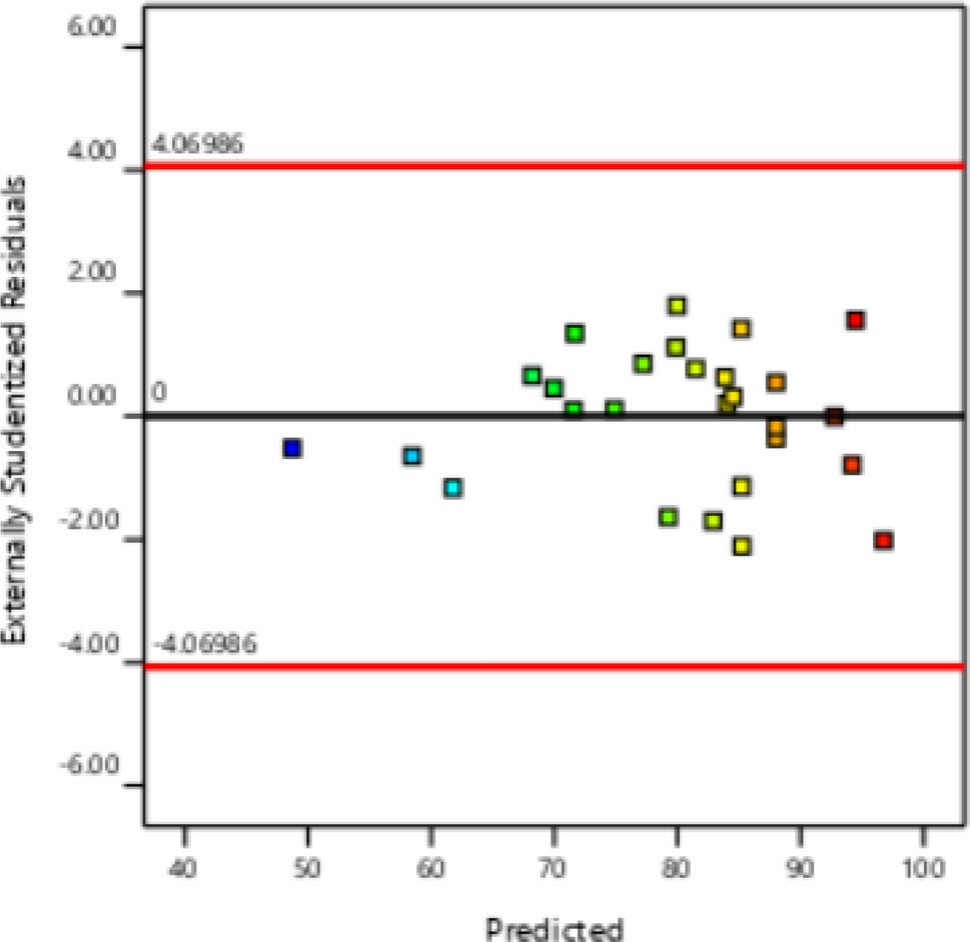
Residuals vs. predicted chart for CV extraction.

**Figure 3:**
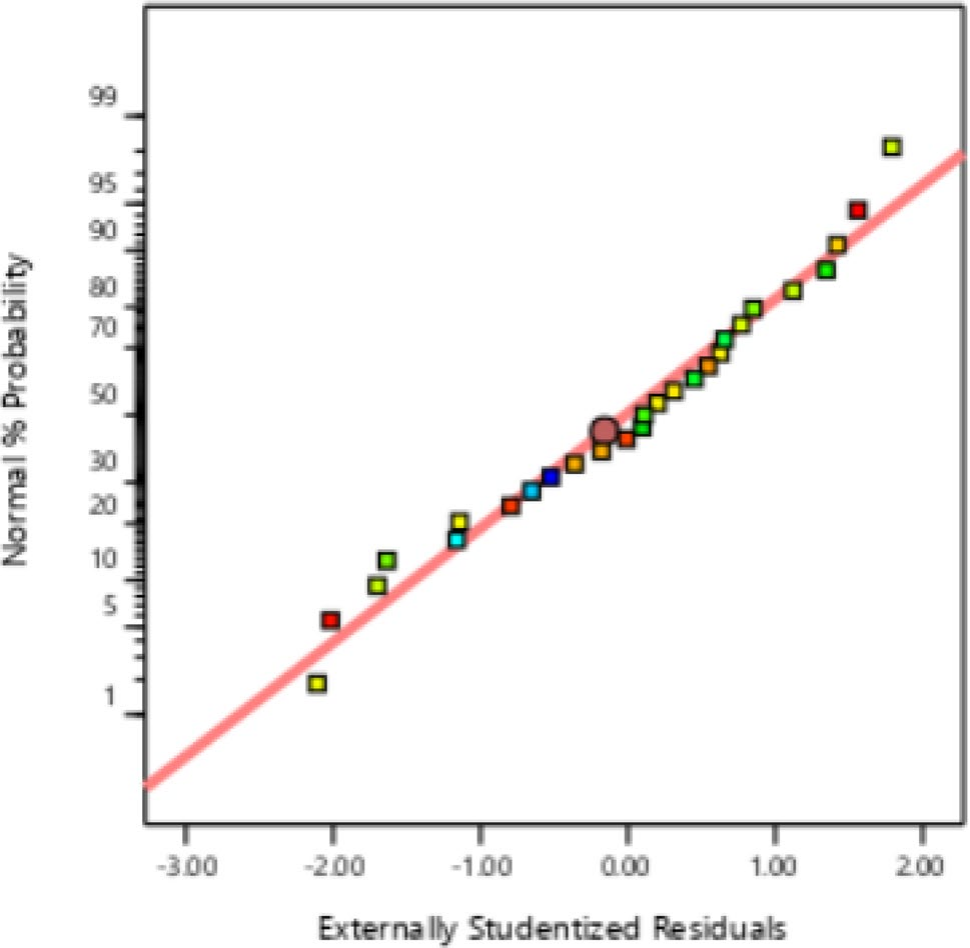
Normal vs. residual probability plot for CV extraction.

**Figure 4:**
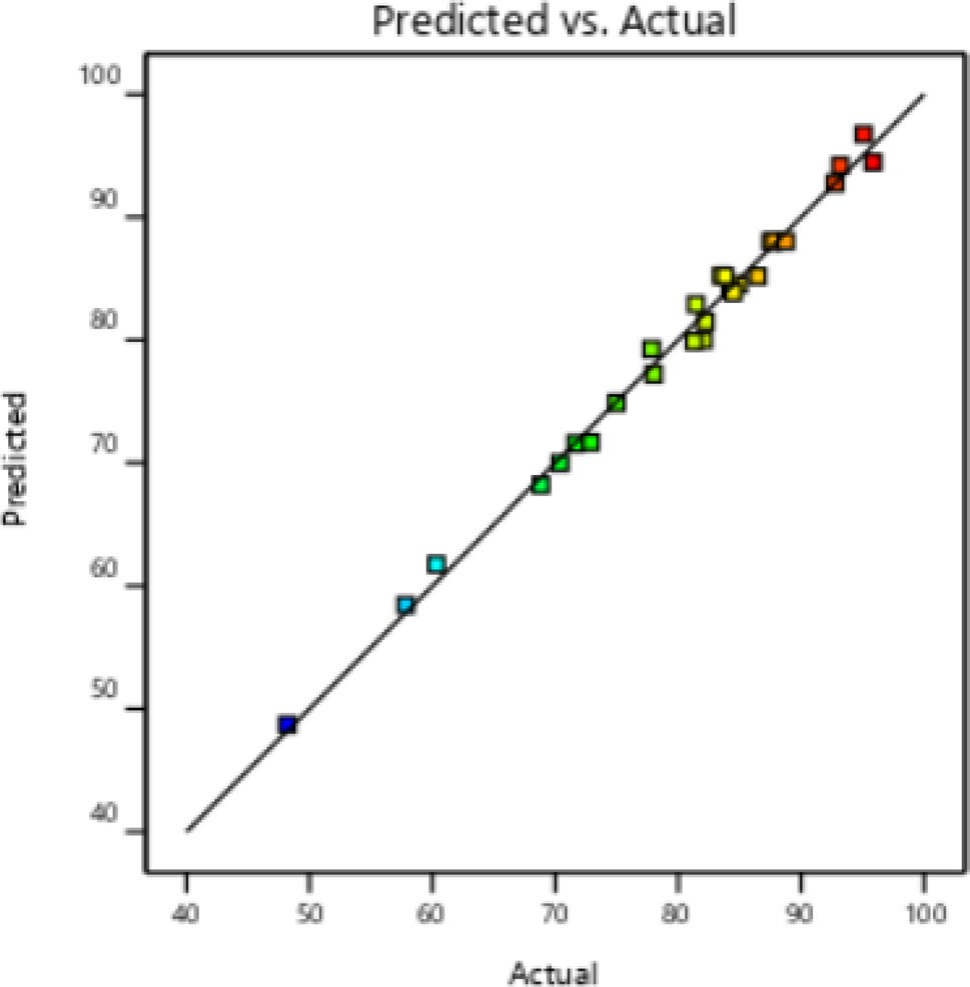
Correspondence between predicted and experimental values of CV extraction efficiency.

The correctness of the model matching was checked by the remaining graph against the predicted values. In this case, all the data must be present within the limit of ± 4.06986, as seen in Fig. 2. The normal probability chart versus the residual value is shown in Fig. 3, which demonstrates the assumption of normality of the experimental data and indicates that the utilized data is near the straight line. Figure 4 displays the graph of the anticipated values for CV extraction versus the actual values. The proper distribution of points around the 45°line indicates that the projected response and the experimental response are in excellent agreement. As a result, the quadratic polynomial model can statistically forecast the volume of CV extraction with accuracy.

## Results and discussion

### Effect of flow rate

**Figure 5:**
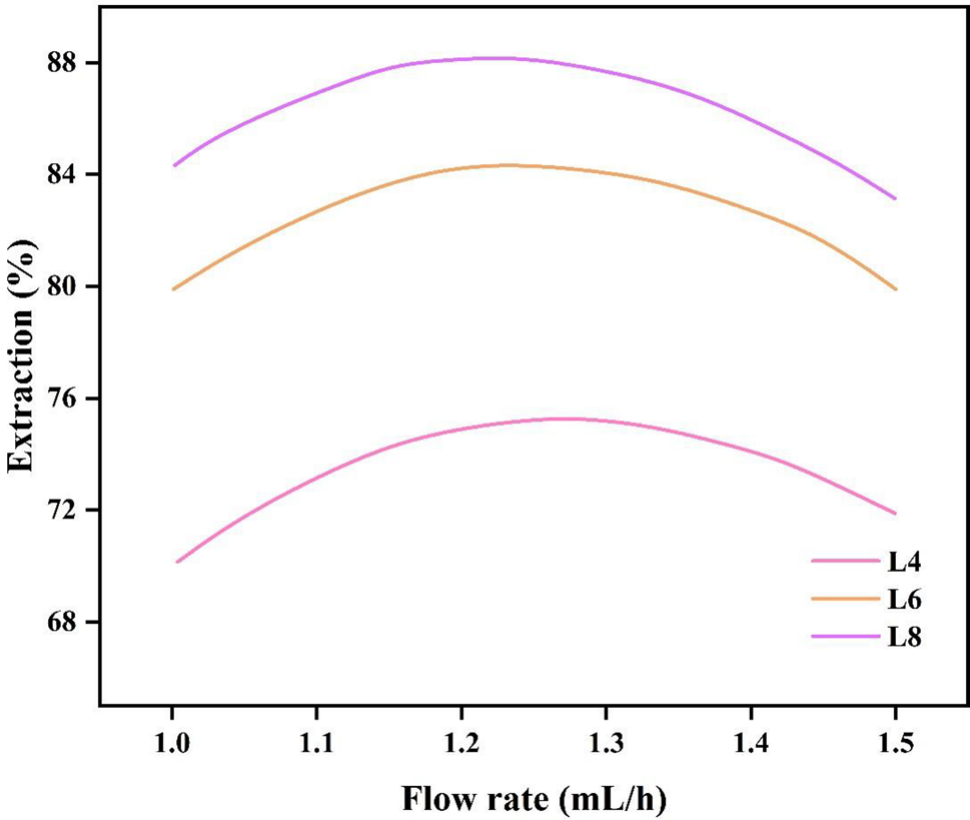
Effect of flow rate on CV extraction efficiency in 3 different lengths.

One of the variables we use to modify the fluid's residence duration in the microchannel is the flow rate. Additionally, the mixing of two phases at the main channel's entry is significantly impacted by the flow rate. The driving force of mass transfer increases with an increase in the two-phase flow rate, and as a result, the extraction efficiency increases, but after a while, the efficiency starts to decrease with an increase in the flow rate. Because at a higher flow rate, the retention time decreases, and the possibility of complete penetration of The aqueous phase does not provide the organic phase in the main channel (Fig. 5). In this study, the flow rates of both phases were determined according to the viscosity of the two phases^21,32^. which was injected with a ratio of 1:1. Because the viscosity of the two phases was close to each other.

### Effect of pH

**Figure 6:**
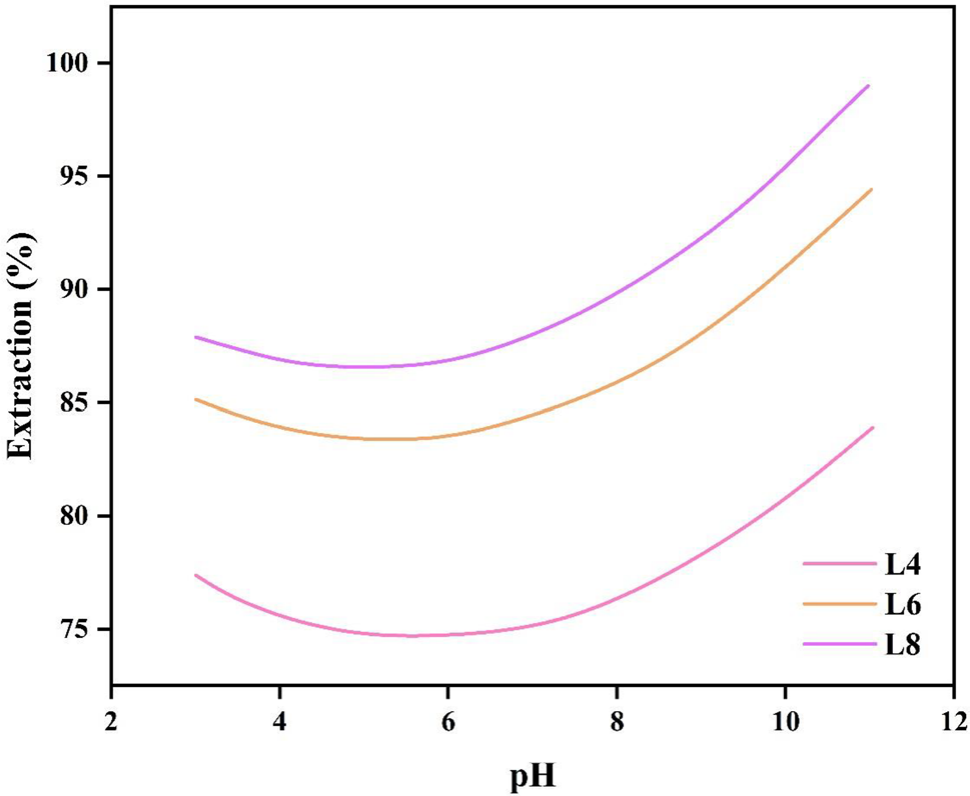
Effect of pH on CV extraction efficiency in 3 different lengths.

The effect of pH of the feed phase in the range of 3–11 on the extraction efficiency was investigated in the condition of 10% v/v of D2EHPA. In Fig. 6, the effect of aqueous phase pH on CV extraction in different lengths of Y-shaped microchannel was investigated. According to the obtained results, the extraction efficiency increases with increasing pH. Because CV is a cationic dye and at high pH, ${OH}^{-}$ forms a colorless complex with CV dye, thus increasing the extraction efficiency.

### Effect of dye concentration

**Figure 7:**
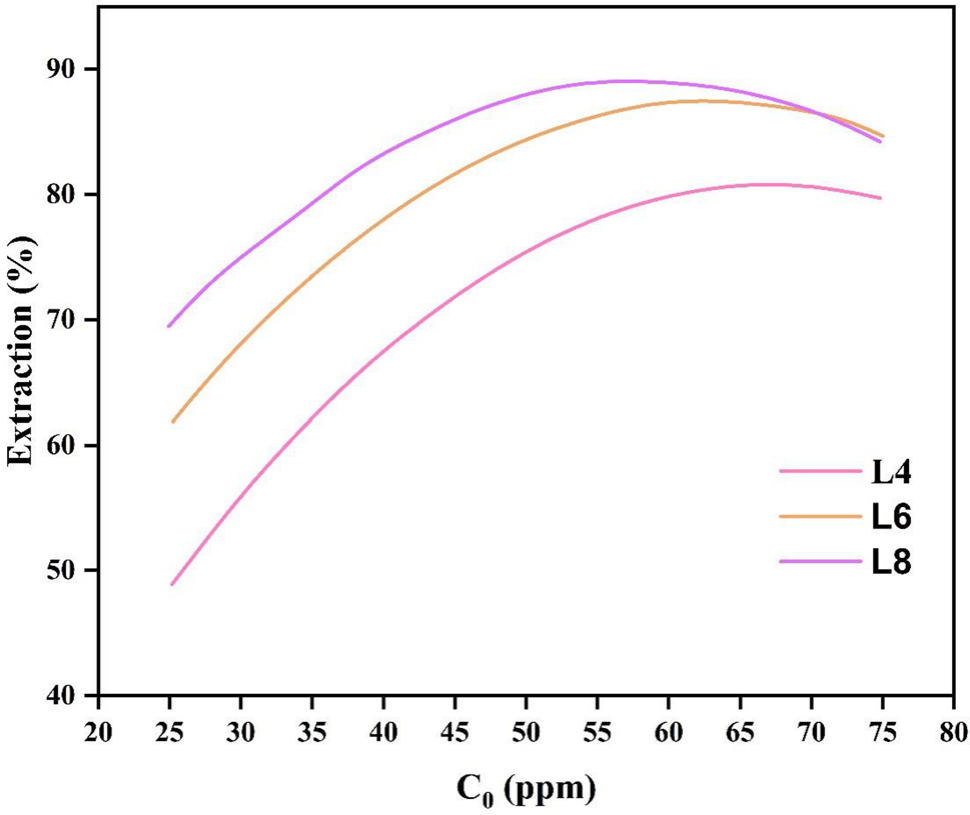
Effect of dye concentration (C_0_) on CV extraction efficiency in 3 different lengths.

Figure 7 shows the effect of input dye concentration on extraction efficiency. In this figure, it is shown that the efficiency increases with the increase of the dye concentration. But with a further increase in dye concentration, the efficiency of the process decreases. Because, initially, the amount of extractant is sufficient and the driving force is high, increasing the concentration of the dye will increase the separation efficiency. The fact that the extractant in the solution cannot react with the excess dye at greater dye concentrations can be used to explain why extraction decreases as dye concentration rises. The tendency of dye molecules may be the cause of the decline in dye removal % with increasing dye concentration. They produce grains with limited penetration by raising the initial concentration of dye, which slows down the pace of dye extraction, a process that is influenced by permeability^33^.

### Effect of microchannel length

**Figure 8:**
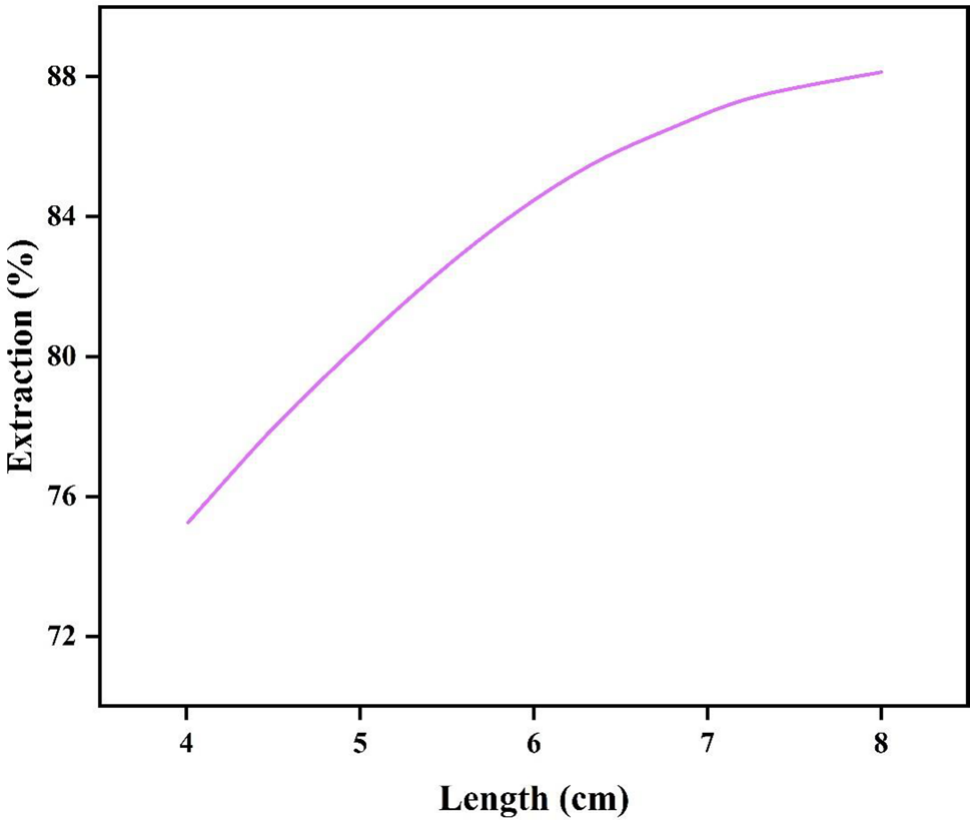
The impact of Y-shaped microchannel length on the effectiveness of CV extraction at pH = 7 and 1.25 mL/h flow rate.

The length of microchannel is one of the significant parameters in improving the extraction performance. As shown in Fig. 8, the extraction efficiency increases with increasing the length of microchannel at fixed values of extractant concentration and initial pH of the feed phase. This trend is due to the increase in the contact time between the two phases with the increase in the residence time due to the increase in the length of microchannel. In parallel flow with a total flow rate of 1.25 mL/h, pH = 7, with the increase in the length of microchannel from 4 to 8 cm, the extraction efficiency increased from 75.34 to 88.01%^34^.

### Analysis of 3D surface curves and contour plots

**Figure 9:**
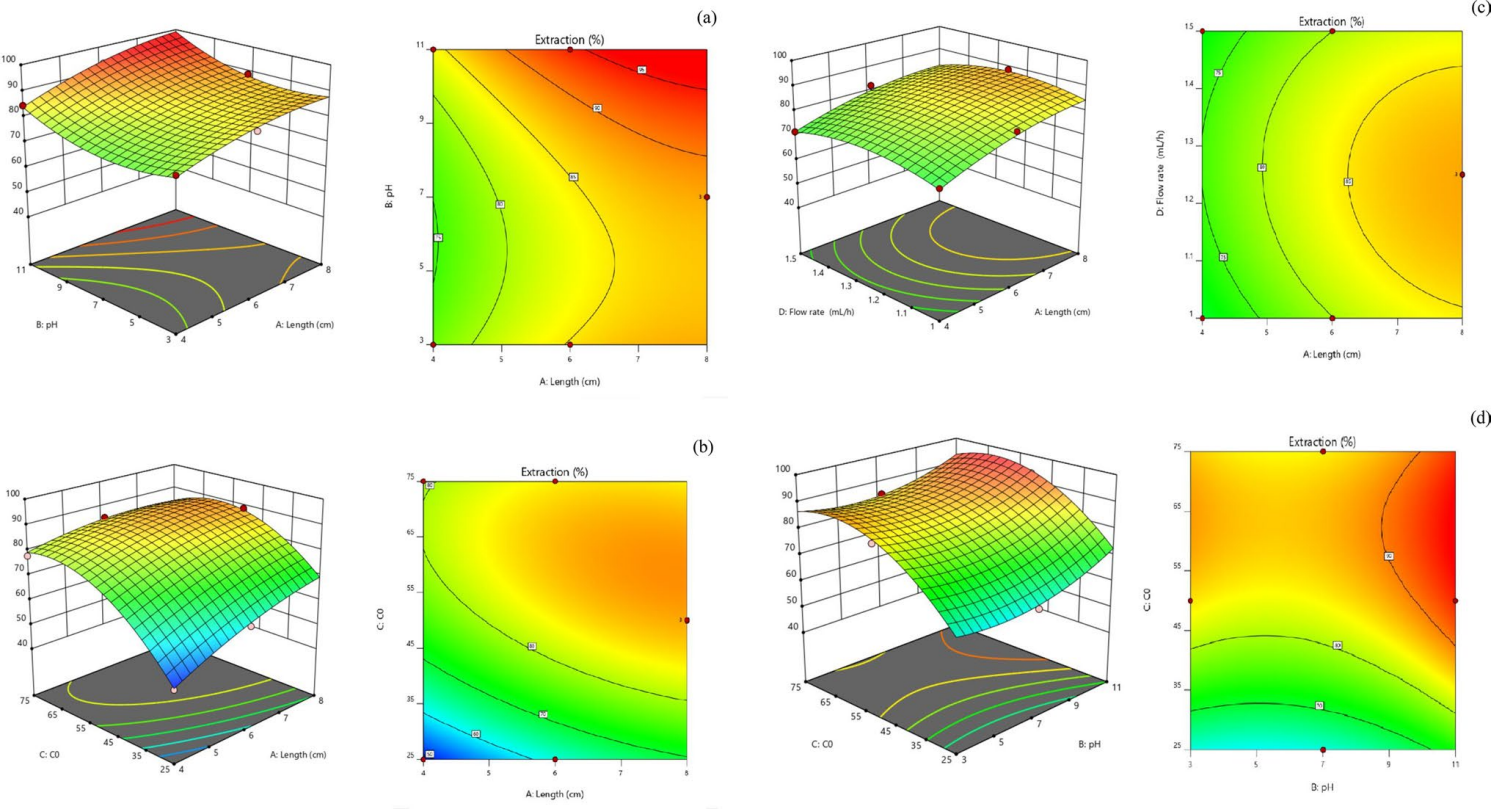

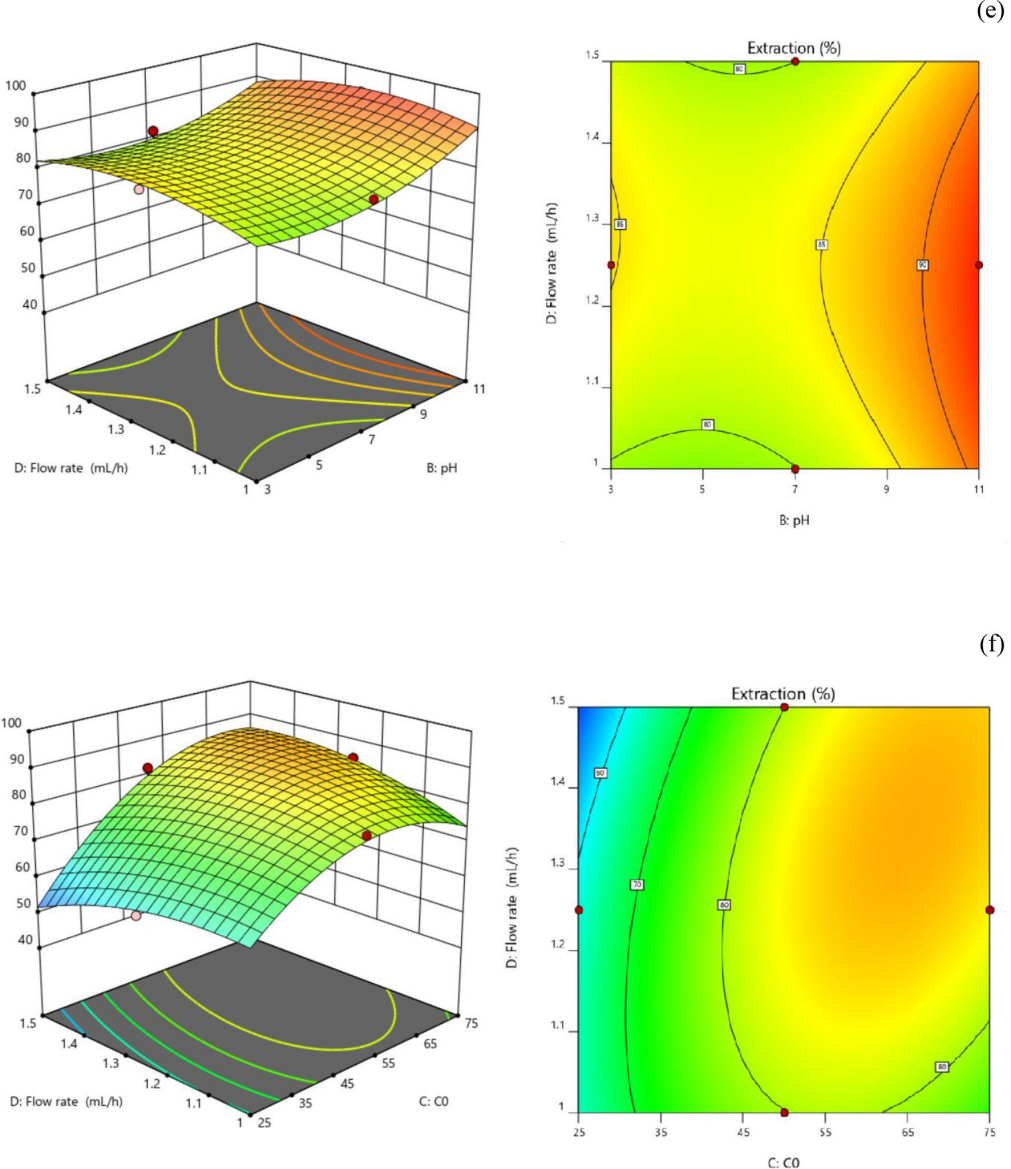
2 and 3D graphs (**a**) Interaction between microchannel length and dye pH (concentration 50 ppm/flow rate 1.25) (**b**) Interaction between microchannel length and input dye concentration pH = 7 and flow rate 1.25 (**c**) the interaction between the length of the microchannel and the flow rate of pH = 7 and the error of 50 ppm (**d**) the interaction between the pH of the dye and the concentration of the dye in the length of 6 cm and the flow rate of 1.25 (**e**) the interaction between the pH of the dye and the flow rate in the length of 6 cm and the concentration of 50 ppm (**f**) the interaction between the dye concentration and the flow rate of the length of 6 cm and pH = 7.

As shown in Fig. 9, 3D response surface curves and contour plots for CV extraction were created to evaluate the impact of each parameter on the response, choose the levels that would yield the highest response, and look at the interactions between the variables.

### Comparison of T and Y-shaped microchannels

**Figure 10:**
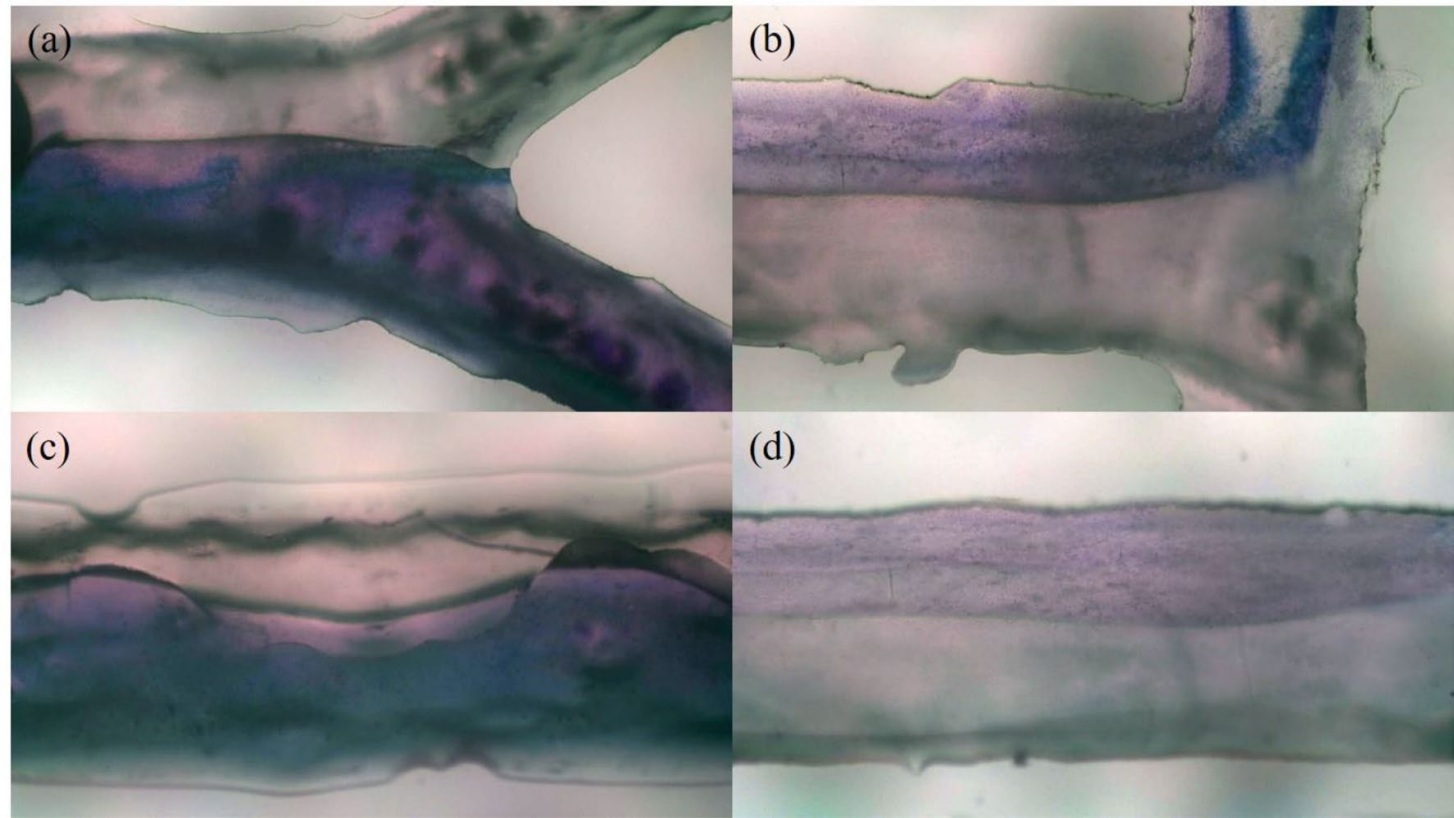
Microscopic images of the studied channels. (**a**) Two-phase contact at the entrance of the main channel in the Y-shaped channel. (**b**) Two-phase contact at the entrance of the main channel in the T-shaped channel. (**c**) Parallel current in the middle of the Y-shaped main channel. (**d**) Parallel current in the middle of the T-shaped main channel.

In this research, the entrance geometry of microchannels in Y and T shapes was investigated. The result showed that for this process, the Y-shaped microchannel has a better performance than the T-shaped microchannel. In the Y-shaped microchannel with an entrance angle of the channel of 56°compared to the T-shaped channel with an entrance angle of 90°, better mixing has been done in the entrance part of the main channel. Also, according to previous studies, the entrance of the main channel has the highest part and influence on the mass transfer between the two phases. Because of this, the speed of mass transfer and mass transfer coefficients and consequently the extraction efficiency increase. Figure 10 shows the microscopic images of the flow difference in Y-shaped and T-shaped channels.

**Figure 11:**
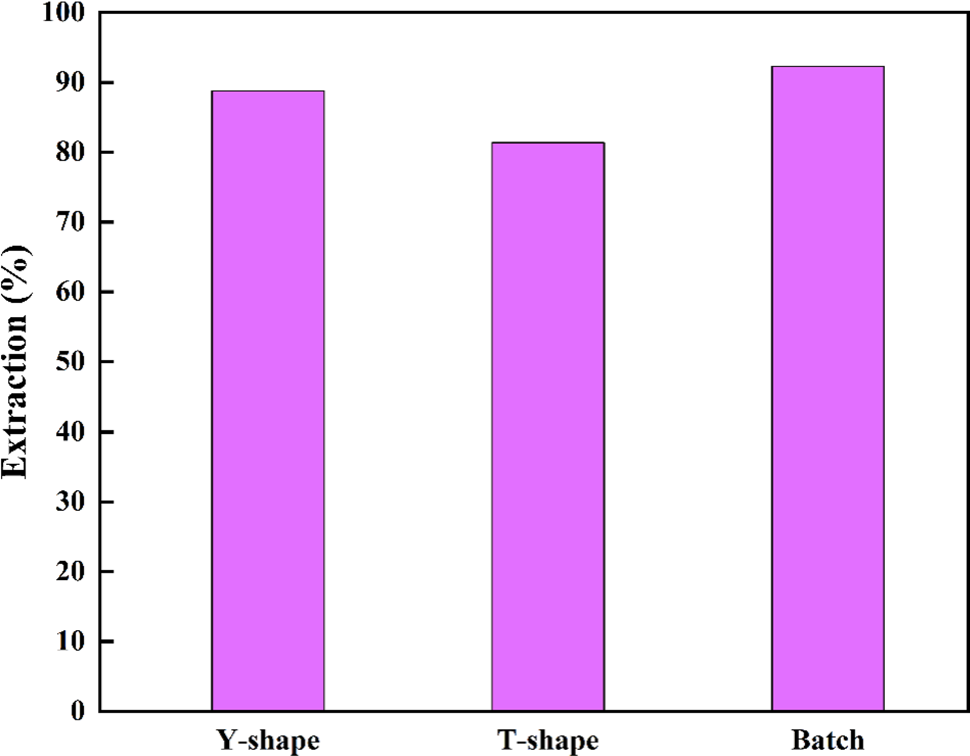
Comparing the efficiency of Y-shaped and T-shaped microchannels and batch extraction process, pH = 7 and C_0_ = 50 ppm.

The residence time in the flow rate of 1, 1.25, and 1.5 mL/h is 17.28, 14.40, and 8.83 (s) respectively. But in the batch extraction process, the time given to separate the two phases was 6 h. Therefore, even though discontinuous extraction has better efficiency under equal conditions, this process has been done for a much longer time, which shows the superiority of the LLE process in its microfluidic (The comparison results of discontinuous and microfluidic systems can be seen in Fig. 11).

### Optimization of LLE microfluidics

Using RSM and looking at how they affected extraction efficiency in the earlier sections, the ideal operating parameters were found. Optimum values of parameters pH, initial concentration, flow rate, and main channel length respectively 10.9, 46.4 ppm, 1.1 mL/h, and 7.6 cm were determined. Under optimal conditions, a value of 97.96% was predicted for extraction efficiency.

An experiment was carried out under ideal circumstances, and the results were compared to what the suggested model anticipated (Table 4). The relative error value is about 0.91%, which confirms the validity and accuracy of this model in predicting the response.

**Table 4:** Optimal values of operating parameters and predicted and experimental values of extraction efficiency.

Parameter	Optimal values		
pH	10.9		
C_0_	46.4 ppm		
Flow rate	1.1 mL/h		
Length	7.6 cm		

Extraction efficiency %	Predict	Actual	Error
	96.97%	95.79%	0.91%

## Conclusion

In this research, solvent extraction of CV using D2EHPA extractant was investigated in parallel flow pattern in Y-Y microchannels. To investigate the effect of inlet geometry and microchannel dimensions on CV extraction efficiency, Y-shaped microchannels with lengths of 4, 6, and 8 cm and width and depth of 400 and 300 μm respectively were made and compared with T-shaped microchannels with an optimal length of 8 cm. The results showed that under the same conditions, the performance of the Y-shaped microchannel is better than the T-shaped microchannel due to the proper contact of the two phases at the entrance of the channel at an angle of 56°. Also, by increasing the length of the microchannel from 4 to 8 cm due to the increase in the contact time between the two phases, the CV extraction efficiency increased from 75.34 to 88.01%. A quadratic polynomial regression model was developed to predict the effect of operating parameters on extraction efficiency. Using ANOVA analysis of variance, the validity and accuracy of the model were investigated. The results showed that the pH of the dye solution phase, flow rate, CV concentration, and microchannel length were obtained as the optimal conditions for extraction, 10.9, 1.1 mL/h, 46.4 ppm, and 7.6 cm, respectively, and the amount of extraction. In this case, 96.97% was achieved. The flow rate has less effect on the extraction efficiency compared to the rest of the investigated parameters. In this study, C_0_ had the greatest effect and flow rate had the least effect on the process.

## Data Availability

All data generated or analyzed data for the experimental part of this study are included in this published article. The data that support the findings of this study are available from the corresponding author, [Ahmad Rahbar-Kelishami], upon reasonable request. Moreover, all other data that support the plots within this paper and other findings of this study are available from the corresponding author upon reasonable request.
